# Relationship between periodontitis and oral cancer: A Mendelian randomization study

**DOI:** 10.6026/973206300221741

**Published:** 2026-03-31

**Authors:** Sreeraj Rajappan, Neethu P Reghu, Shilpa Mathew, Bindu R Nayar, Fathima P.H

**Affiliations:** 1 Department of Periodontics, Government Dental College, Alappuzha, Kerala, India; 2 Department of Periodontics, Government Dental College, Kottayam, Kerala, India; 3 Department of Periodontics, Government Dental College, Thiruvananthapuram, Kerala, India

**Keywords:** Periodontitis, aggressive periodontitis, mouth neoplasms, oropharyngeal neoplasms, causality, Mendelian randomization analysis

## Abstract

Previous observational studies have found a positive association between periodontitis and oral cancer; however, the causal role of periodontitis in oral cancer has not yet been determined. Mendelian randomization (MR) studies are considered superior to traditional observational studies for causal inferences. Therefore, it is of interest to examine the potential causal link between periodontitis and oral cancers using MR analysis. Data were retrieved from the NHGRI-EBI GWAS Catalogue within MR-Base using the "TwoSampleMR" R package. The analysis employed the inverse variance method, with sensitivity analysis using MR-Egger, weighted median and mode approaches. MR analysis showed no association between periodontitis and oral cancer. However, aggressive periodontitis showed significance (p = 0.001), with an odds ratio of 1.39 (95% CI: 1.14-1.69), indicating causality.

## Background:

Periodontitis is a chronic inflammatory condition characterized by loss of periodontal tissue support through periodontal pockets, loss of clinical attachment and radiographic bone loss [1]. Its global prevalence ranges from 20% to 50% [2]. Aggressive periodontitis is an uncommon but severe form of periodontitis, with a global burden of approximately 1.6% [3]. Oral and oropharyngeal cancers (hereafter referred to as oral cancers) are among the most common cancers worldwide. It is a highly morbid condition with high mortality, characterized by late detection, poor prognosis, absence of specific biomarkers and costly treatment [4]. Observational studies exploring the relationship between periodontitis and oral cancer have found a positive association [5, 6], with a pooled odds ratio of 3.53 [7]. This association may be causal, as persistent inflammation in periodontitis can cause malignant transformation of the affected oral epithelium [8]. The findings of observational studies have limited value for causal inference because of potential biases and unmeasured confounders. In addition, a randomized controlled trial (RCT), the most reliable evidence for determining causal relationships, is neither ethically permissible nor feasible. Mendelian randomization (MR) can overcome these problems by using single nucleotide polymorphisms (SNPs) as proxies for environmental exposures. Based on Mendel's laws of segregation and independent assortment, SNPs are allocated independently of confounders [9], achieving effects similar to those of RCT randomization. MR estimates causal effects under specific assumptions [10]. The NHGRI-EBI GWAS catalogue in the MR-Base database was used as the data source [11]. SNP associations for periodontitis and aggressive periodontitis as exposures were derived from the GWAS of 3915 (Teumer *et al.* 2013) [12] and 7687 individuals (Munz *et al.* 2017) [13], respectively. Genetic association estimates of SNPs with oral cancer as an outcome were obtained from a GWAS of 5425 individuals (Lesseur *et al.* 2016) [14]. Details of these GWASs are given in Table 4 (see PDF). Therefore, it is of interest to report the causal link between periodontitis and aggressive periodontitis with oral cancers by means of MR analysis using this data source.

## Materials and Methods:

This study used a two-sample Mendelian randomization (2SMR) strategy to infer causal relationships between the phenotypes (Figure 1 - see PDF). To decrease the possible bias from population stratification, MR analyses were limited to people of European descent. The study protocol and details were not preregistered. This study was reported based on the recommendations of the STROBE-MR and "Guidelines for performing Mendelian randomization investigations." This study was conducted using publicly available summary data from genome-wide association studies (GWAS). Each GWAS included in this study received approval from an ethical review board and obtained informed consent, as detailed in the original manuscripts. Institutional Review Board and Ethics Committee approvals were obtained for this study. SNPs associated with exposure and with a p-value < 1 x 10^-5^ were chosen as instrumental variables. Clumping was used to determine SNPs with linkage disequilibrium (LD) with other SNPs. To ensure consistency, the impact of SNPs on both exposure and outcome was harmonized so that the influence of an SNP on exposure matched the influence of that SNP on the outcome for the same allele. An attempt was made to align the strands of palindromic SNPs. From the candidate instrumental variables, SNPs with LD with other SNPs and SNPs with incompatible alleles were removed.

## Assumptions for valid instrumental variables:

Three essential assumptions define valid instrumental variables: First, the genetic marker must be linked to the exposure. Second, the genetic marker should remain independent of the outcome when considering the exposure and all confounders, whether measured or not, in the exposure-outcome relationship (this is known as exclusion restriction). Third, the genetic marker must be independent of any factors, both measured and unmeasured, that could confound the exposure-outcome relationship, meaning that it should influence the outcome solely through exposure [15] (Figure 2 - see PDF). Exposure and outcome variables were dichotomized as present or absent in the original studies. The R package "Two Sample MR" [16] version 0.5.6 using RStudio version 2023.03.0 (R version 4.2.2) was used for statistical analysis. The MR estimator used for individual SNPs was the Wald ratio, which was derived by dividing the effect estimate for the SNP-outcome association by the coefficient of the SNP exposure association. The inverse-variance weighted (IVW) MR method was used as the primary analysis method to derive the causal estimate. Previous reports of associations between genetic instruments and traits potentially confounding the exposure-outcome or SNP-outcome association were explored using the online Phenol Scanner version 2.0 [17]. Mendelian randomization-Egger (MR-Egger), weighted median, simple mode and weighted mode were used for sensitivity analyses. Leave-one-out validation was performed to assess the sensitivity of each instrumental variable.

## Results and Discussion:

The IVW analysis did not find evidence of a causal link between periodontitis (in general) and oral cancer risk (Table 1 - see PDF). This finding contrasts with those of most previous observational studies, which have found a positive association between periodontitis and oral cancer. The spurious relationship seen in previous studies may be due to the effect of unmeasured confounders, such as poor nutritional status [18] or common genetic risk factors; incomplete adjustment for or failure to exclude important known risk factors, such as smoking [19]; or systematic bias, such as selective survival and Berksonian bias. However, this concurs with the findings of Xio *et al.* [20] and Sheng *et al.* [21], who found no association between periodontitis and oral cancer using the MR technique. Nonetheless, the IVM revealed a significant causal effect of aggressive periodontitis on oral cancer, with a p-value of 0.001 and an odds ratio of 1.39 (95% CI: 1.14-1.69). Although this is a small causal effect, considering the seriousness of the outcome, it may be important and clinically significant, especially in patients with coexisting risk factors. This is in contrast to the findings of Xio *et al.* [20], who found a negative association between acute periodontitis and oral cancer using the IVM method. However, other methods, such as MR-Egger, weighted median, simple mode and weighted mode, did not show significant causal effect estimates for aggressive periodontitis and oral cancer (Table 1 - see PDF). Tests for heterogeneity using the Q statistics for periodontitis and aggressive periodontitis as exposures yielded p-values that were not statistically significant, indicating adequate homogeneity of individual SNP effects (Table 2 - see PDF). Single SNP MR Analysis showed that all five selected instruments were positively associated with oral cancer. Of these, two associations (for SNPs rs2978951 and rs2070901) were found to be statistically significant (Figure 3 - see PDF). Although the intercept term from the MR-Egger regression was 0.182, which did not significantly differ from zero (Table 3 - see PDF), the causal estimate from the MR-Egger analysis was not significant (Table 1 - see PDF). The scatter plot of the SNP effect on aggressive periodontitis and oral cancer showed an absence of a dose response, indicating the possibility of horizontal directional pleiotropy (Figure 4 - see PDF). The MR leave-one-out sensitivity analysis for aggressive periodontitis and oral cancer showed persistence of association even after the removal of these SNPs (Figure 5 - see PDF).

All SNPs associated with exposure to aggressive periodontitis had a p-value ≤ 1 x 10^-6^. This satisfies the first assumption (relevance assumption) of a valid instrument. Exploration of previous studies associated with aggressive periodontitis SNPs using PhenoScanner revealed that rs2978951 was associated with the percentage of monocytes and basophils among white cells, as well as monocyte and basophil counts [22]. The variant rs2070901 has been linked to allergic conditions such as asthma, hay fever and eczema [23]. This may violate the third assumption of a valid instrument because allergic conditions are suspected to influence oral cancer [24]. Thus, the results of the sensitivity analysis are inconclusive. This potential causal connection is biologically plausible because aggressive periodontitis is associated with periodontal pathogens such as *Aggregatibacter actinomycetemcomitans* and *Porphyromonas gingivalis*, both of which are suspected to be carcinogenic [25, 26- 27]. An important virulence toxin produced by *Aggregatibacter actinomycetemcomitans* is the cytolethal distending toxin (CDT), which contains a nuclease motif homologous to DNase I. The nuclease activity of CDT is involved in the formation of DNA double-strand breaks (DSBs) in affected mammalian cells [28]. DSB formation in host cells causes genomic instability, which can subsequently increase the risk of carcinogenesis. *Porphyromonas gingivalis* can promote the development of oral cavity and digestive tract cancers by inducing epithelial-to-mesenchymal transition, activating metalloproteinase-9 and interleukin-8, accelerating cell cycling and suppressing apoptosis [29]. The protease generated by *Porphyromonas gingivalis* breaks down the extracellular matrix, damages the host's epithelial tissue, severely impairs the host's immune system and may ultimately lead to the initiation and advancement of tumors [30]. The early onset and persistence of periodontopathogens associated with aggressive periodontitis, even after treatment in the oral and oropharyngeal regions [31], may allow for the long induction period required for the development of oral cancer. This MR study used GWAS data from studies involving participants of European descent. MR studies using GWAS data involving other populations can be performed to ensure the generalizability of the findings. A multivariable Mendelian randomization approach (factorial Mendelian randomization), which involves multiple genetic variants linked to various measured risk factors, such as aggressive periodontitis and allergic conditions, can be used to simultaneously determine the causal effects of each of these risk factors. Basic and clinical research, including animal studies, should further explore the role of aggressive periodontitis and associated bacteria, particularly *Aggregatibacter actinomycetemcomitans* in the pathogenesis of oral cancer. Because of the potentially increased risk of oral cancer, patients with aggressive periodontitis should be strictly monitored, especially those with coexisting lifestyle risks, such as tobacco use and alcohol consumption.

## Conclusion:

We show no evidence of a causal role for periodontitis (in general) in oral cancer. However, there is some evidence that a severe but relatively uncommon form of periodontitis, namely aggressive periodontitis, may be causally related to oral cancer. Evidence from multiple sources, such as factorial MR studies and basic, animal and experimental research exploring biological pathways, can provide evidence for a potential causal connection between aggressive periodontitis and oral cancer.

## Declaration on publication ethics:

The author's state that they adhere with COPE guidelines on publishing ethics as described elsewhere at https://publicationethics.org/. The authors also undertake that they are not associated with any other third party (governmental or non-governmental agencies) linking with any form of unethical issues connecting to this publication. The authors also declare that they are not withholding any information that is misleading to the publisher in regard to this article.

## License statement:

This is an Open Access article which permits unrestricted use, distribution and reproduction in any medium, provided the original work is properly credited. This is distributed under the terms of the Creative Commons Attribution License.

## Comments from readers:

Articles published in BIOINFORMATION are open for relevant post publication comments and criticisms, which will be published immediately linking to the original article without open access charges. Comments should be concise, coherent and critical in less than 1000 words.
